# Serum Lactate Levels and Their Correlation With Hospital Outcomes in ICU Patients With Shock: A Cross-Sectional Study at a Tertiary Care Center

**DOI:** 10.7759/cureus.86564

**Published:** 2025-06-22

**Authors:** Meghana R Baser, Rajesh Ganta, Charan Neeradi, Sakthivadivel Varatharajan

**Affiliations:** 1 General Medicine, Manik Hospital and Research Center, Chhatrapati Sambhajinagar, IND; 2 General Medicine, CARE Hospital, Hyderabad, IND; 3 General Medicine, All India Institute of Medical Sciences (AIIMS) Bibinagar, Hyderabad, IND

**Keywords:** cardiogenic shock, hospital outcomes, intensive care unit (icu), septic shock, serum lactate, shock, shock index, tertiary care center

## Abstract

Background and aim: Shock is a life-threatening state characterized by inadequate tissue perfusion to meet metabolic demand, leading to organ failure and death. Serum lactate, a byproduct of anaerobic metabolism, has been identified as a significant biomarker for assessing shock severity and predicting outcomes. This study aimed to evaluate the correlation between serial serum lactate levels and hospital outcomes in patients with shock admitted to the ICU at Manik Hospital.

Methods: A prospective observational study was conducted, including 124 adult patients diagnosed with shock and admitted to the ICU between April 2023 and December 2023. Serum lactate levels were measured at admission and 24 hours later. Outcomes assessed included mortality, mechanical ventilation, ICU stay duration, and shock index. Data were analyzed using descriptive and inferential statistics, including Pearson correlation and receiver operating characteristic (ROC) curves.

Results: The study participants had a mean age of 50.7 years, with hypovolemic shock being the most common condition, affecting 61.3% (n=76). A moderate positive correlation was found between serum lactate levels and the shock index at both admission (r=0.273) and 24 hours (r=0.293) (p<0.001). Elevated lactate levels at both time points were significantly associated with increased mortality and the need for mechanical ventilation (p<0.001). At admission, lactate predicted mortality with an area under the curve (AUC) of 0.765, which increased to 0.848 at 24 hours. Lactate also showed good predictive power for the need for mechanical ventilation, with AUC values of 0.737 at admission and 0.664 at 24 hours.

Conclusions: Elevated serum lactate levels, both at admission and 24 hours, were identified as strong predictors of mortality, mechanical ventilation, and prolonged ICU stay in shock patients. Serial lactate measurements were found to provide valuable prognostic information, aiding in early risk stratification and personalized patient management. Further research was recommended to validate these findings across multiple centers and explore the role of lactate clearance in predicting recovery.

## Introduction

Shock is a life-threatening state marked by inadequate tissue perfusion to meet metabolic demand, which, if untreated, leads to cellular hypoxia and dysfunction of multiple organ systems [1]. It is a significant cause of admission to intensive care units (ICUs) worldwide, with timely identification and management being crucial for preventing organ failure, prolonged ICU stays, and mortality. Shock can arise from various etiologies, including hypovolemic, septic, cardiogenic, and obstructive shock, all of which share the common pathophysiological feature of impaired tissue perfusion and oxygen delivery [1,2]. Without proper intervention, shock can rapidly progress to irreversible organ failure and death, underscoring the need for early diagnosis and management.

Serum lactate, a byproduct of anaerobic metabolism, has gained considerable attention as a reliable biomarker in assessing shock. When tissues experience inadequate oxygen delivery, they switch to anaerobic metabolism, leading to increased lactate production. Elevated serum lactate levels indicate tissue hypoxia and metabolic distress, which are associated with poor clinical outcomes [3]. Due to its association with worse outcomes, lactate measurements are increasingly used to guide clinical management, monitor the progression of shock, and assess treatment efficacy.

Numerous studies demonstrate the strong correlation between elevated lactate levels and adverse outcomes in shock patients, particularly in septic shock [4-10]. Elevated lactate levels have been linked to a higher likelihood of requiring intensive care, including mechanical ventilation and prolonged ICU stays, and have proven predictive value for mortality [8]. While substantial evidence supports the prognostic value of serial serum lactate measurements in septic shock, their utility in predicting morbidity and hospital outcomes across all shock etiologies remains less well established. Many studies have focused on single lactate measurements, neglecting to evaluate lactate dynamics over time and their relationship to clinical outcomes.

Additionally, existing research is limited by several important factors. First, most studies have focused on specific types of shock, such as septic shock, limiting the generalizability of their findings to other forms of shock, including hypovolemic and cardiogenic shock. Second, most studies have concentrated on lactate as a predictor of mortality, with fewer investigating how lactate dynamics affect other important clinical outcomes, such as the need for mechanical ventilation or the duration of ICU and hospital stays. Lastly, the relationship between lactate levels and the shock index, a key clinical parameter reflecting the severity of shock, has not been adequately explored in the context of serial lactate measurements.

This study aimed to address these gaps by examining the correlation between serial serum lactate levels and hospital outcomes in patients admitted to the ICU with shock at a tertiary care hospital in western India. By evaluating the association between lactate levels and various clinical outcomes, including mortality, mechanical ventilation, ICU stay, and shock index, the study sought to clarify the predictive value of lactate measurements over time. Specifically, it assessed the accuracy of baseline and 24-hour serum lactate levels in predicting mortality and indicators of clinical severity, such as need for mechanical ventilation, ICU stay, and shock index, while also exploring their relationship with the shock index to better understand the utility of lactate measurements in clinical decision-making.

## Materials and methods

This was a prospective observational study conducted at a tertiary care center in Maharashtra, which is in the western part of India. The study was initiated after approval from the Seth Nandlal Dhoot Hospital Institutional Ethics Committee (#631). Patients admitted to the ICU with shock between April 2023 and December 31, 2023, were included in the study. Patients aged >18 years with a clinical diagnosis of shock, defined as persistent hypotension (mean arterial pressure {MAP} <65 mmHg or SBP <90 mmHg) after adequate resuscitation, were included in the study [11]. Clinical examination findings such as cold, clammy extremities and weak pulses were noted to support the diagnosis of hypoperfusion in vasoconstricted states, while in cases of distributive shock (e.g., septic shock), warm extremities and vasodilation were observed. These findings were used to aid classification of shock type. Patients who were pregnant or lactating, immunocompromised, or on medications altering lactate metabolism (e.g., metformin) were excluded. The sample size was calculated based on the standard deviation (SD) of serum lactate levels (mean=3.77, SD=1.42), with a desired confidence level of 95%, resulting in a sample size of 124 patients [12]. Upon admission, detailed history-taking and physical examinations were performed to determine the type of shock, and routine baseline blood investigations, along with additional tests as needed (e.g., ECG, 2D echocardiography {ECHO} for cardiogenic shock, and blood and urine cultures for septic shock), were conducted. Serum lactate levels were measured at admission and repeated 24 hours later. Treatment was optimized according to clinical findings, and management protocols for different shock types (cardiogenic, septic, hypovolemic) were followed. Outcomes were defined by the length of ICU stay, days on mechanical ventilation, total hospital stay, and the need for additional medical interventions. The Shock Index (SI), calculated as the ratio of heart rate (HR) to systolic blood pressure (SBP), was also used to assess the severity of shock. Data were analyzed using SPSS version 24.0 (IBM Corp.: Armonk, NY), with descriptive statistics (mean, median, standard deviation) for quantitative variables and proportions for categorical variables. The chi-square test and unpaired t-test were used to assess the significant association between serum lactate levels and patient outcomes. The Pearson correlation coefficient was used to determine correlations between serum lactate levels and the Shock Index, and receiver operating characteristic (ROC) curves assessed the accuracy of serum lactate levels in predicting outcomes. A p-value of <0.05 was considered statistically significant.

## Results

A total of 124 participants were enrolled in the study, comprising 72 males (58%) and 52 females (42%). The mean age of the participants was 50.69±15.79 years. Clinical, demographic, and laboratory characteristics are summarized in Table 1.

**Table 1:** Demographic, clinical and lab characteristics of participants. *Values are expressed as mean±standard deviation. SBP: systolic blood pressure; DBP: diastolic blood pressure

Characteristics	Total population=124, n (%)
Mean age (years)	50.69±15.79*
Male	72 (58%)
HR (per minute)	106.66±18.68*
SBP (mmHg)	88.40±20.25*
DBP (mmHg)	65.5±19.97*
BMI (kg/m^2^)	24.43±3.4*
Shock index (SI)	1.28±0.41*
Mechanical ventilation requirement	65 (52.4%)
Shock type	
Cardiogenic	10 (8.1%)
Hypovolemic	76 (61.3%)
Septic	38 (30.6%)
Serum lactate	
At 0 hours (mmol/L)	4.64±3.82*
At 24 hours (mmol/L)	6.81±3.82*
WBC (×10^3^/µL)	13.77±6.26*
ICU stay (days)	3.10±2.74*
Number of days on ventilator	1.04±1.26*
Hospital stay (days)	3.22±2.93*
Mortality	35 (28.2%)

Among the types of shock observed, hypovolemic shock was the most common, affecting 76 participants (61.3%). Septic shock was present in 38 participants (30.6%), while cardiogenic shock was seen in 10 participants (8.1%). The mean heart rate (HR) at presentation was 106.66±18.68 beats per minute. The mean systolic and diastolic blood pressures (SBP and DBP) were 88.40±20.25 mmHg and 65.50±19.97 mmHg, respectively. The average body mass index (BMI) was 24.43±3.40 kg/m^2^, and the mean shock index (SI) was 1.28±0.41. Mechanical ventilation was required in 65 participants (52.4%).

The mean serum lactate levels were 4.64±3.82 mmol/L at admission and 6.81±3.82 mmol/L at 24 hours. The mean white blood cell (WBC) count was 13.77±6.26 × 10^3^/µL. The median duration of ICU stay was 3.10±2.74 days, with a mean hospital stay of 3.22±2.93 days. The mean number of days on mechanical ventilation was 1.04±1.26 days. Overall, mortality was observed in 35 participants (28.2%).

Association of serum lactate with mechanical ventilation and mortality

Participants who experienced mortality had significantly higher mean serum lactate levels at both admission and 24 hours post-admission compared to survivors. At admission, the mean lactate level among non-survivors was 7.68±4.92 mmol/L, while it was 3.46±2.45 mmol/L among survivors (p=0.001). Similarly, at 24 hours, the mean lactate level increased to 10.17±3.59 mmol/L in non-survivors, compared to 5.56±3.07 mmol/L in survivors (p=0.001) (Table 2).

**Table 2:** Association of serum lactate at admission and 24 hours with mortality and mechanical ventilation requirement. Data are presented as mean±standard deviation. Comparisons between groups were performed using the independent samples t-test. The t-value represents the calculated test statistic used to assess the significance of differences in mean lactate levels between the respective groups.

Variables		Lactate at admission (mean±SD) (mmol/L)	Lactate at 24 hours (mean±SD) (mmol/L)	p-Value		t-Value	
				Lactate at admission	Lactate at 24 hours	At admission	At 24 hours
Mortality	Yes (n=35)	7.67±4.92	10.17±3.59	0.001	0.001	4.84	6.71
	No (n=89)	3.45±2.45	5.55±3.07				
Mechanical ventilation requirement	Yes (n=65)	5.94±4.07	7.84±3.91	0.001	0.003	4.28	3.12
	No (n=59)	3.22±2.96	5.78±3.44				

A similar trend was observed with respect to mechanical ventilation. Participants who required mechanical ventilation had significantly higher lactate levels than those who did not. At admission, the mean lactate level was 5.94±4.07 mmol/L in ventilated patients, compared to 3.22±2.96 mmol/L in non-ventilated patients (p=0.001). At 24 hours, the mean lactate level further increased to 7.84±3.91 mmol/L in ventilated individuals, compared to 5.78±3.44 mmol/L in those not requiring ventilation (p=0.003) (Table 2).

**Figure 1:**
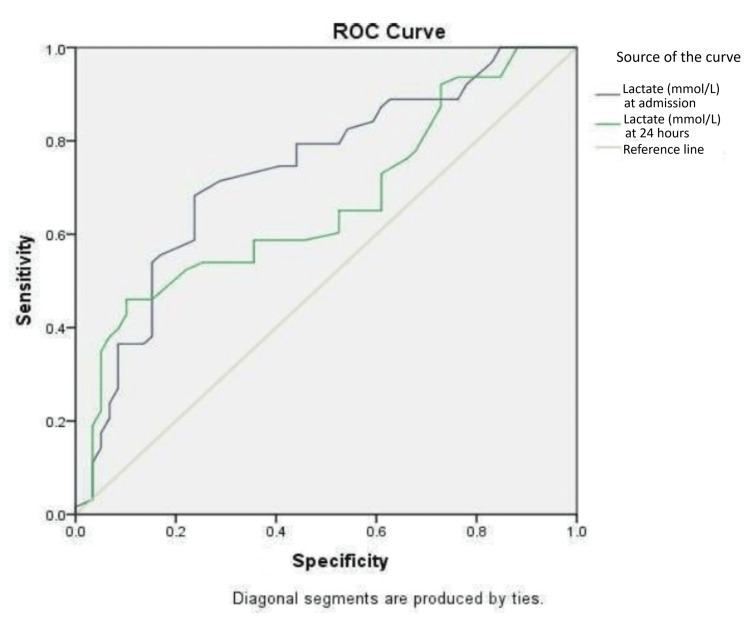
Diagnostic accuracy of serum lactate in predicting the need for mechanical ventilation. Receiver operating characteristic curves of the serum lactate levels at admission and 24 hours. ROC: receiver operating characteristic

The diagnostic accuracy of serum lactate in predicting the need for mechanical ventilation was assessed using receiver operating characteristic (ROC) analysis. At admission, the area under the curve (AUC) was 0.737 (SE=0.046, p<0.001; 95% CI: 0.647-0.826), indicating good discriminatory ability. At 24 hours, the AUC declined to 0.664 (SE=0.049, p=0.002; 95% CI: 0.567-0.761) (Table 3 and Figure 1).

**Table 3:** Accuracy of serum lactate in predicting mechanical ventilation requirement. The area under the ROC curve (AUC) was calculated to assess the prognostic performance of lactate levels in predicting mechanical ventilation requirement. ROC: receiver operating characteristic

Time point	AUC	SE	p-Value	95% Confidence interval
At admission	0.737	0.046	<0.001	0.647-0.826
At 24 hours	0.664	0.049	0.002	0.567-0.761

**Figure 2:**
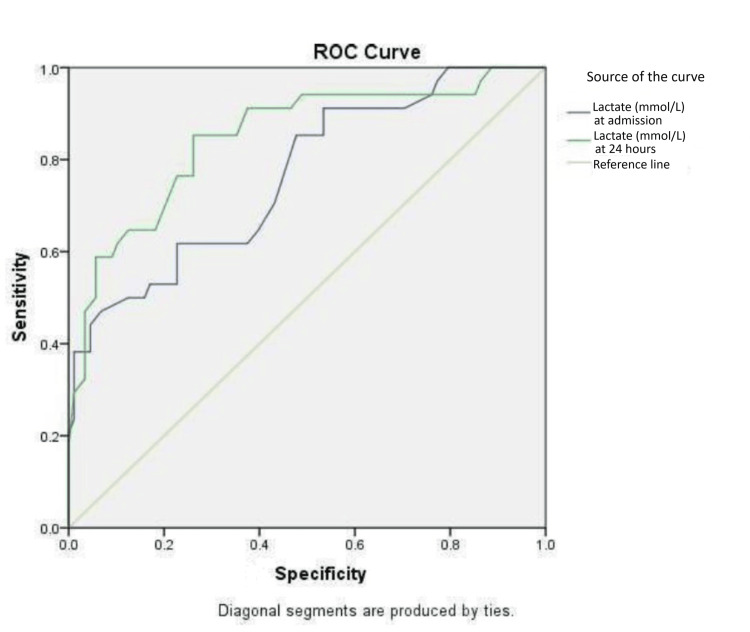
The diagnostic accuracy of serum lactate in predicting mortality. Receiver operating characteristic curves of the serum lactate at admission and 24 hours. ROC: receiver operating characteristic

For predicting mortality, the AUC for serum lactate at admission was 0.765 (SE=0.049, p<0.001; 95% CI: 0.669-0.861). At 24 hours, the predictive ability improved significantly with an AUC of 0.848 (SE=0.042, p<0.001; 95% CI: 0.765-0.931), suggesting excellent prognostic value (Table 4 and Figure 2).

**Table 4:** Accuracy of serum lactate in predicting mortality. The area under the ROC curve (AUC) was calculated to assess the prognostic performance of lactate levels in predicting mortality. ROC: receiver operating characteristic

Time point	AUC	SE	p-Value	95% Confidence interval
At admission	0.765	0.049	<0.001	0.669-0.861
At 24 hours	0.848	0.042	<0.001	0.766-0.929

The relationship between shock index (SI) and serum lactate levels was evaluated using Pearson correlation analysis. At admission, the correlation coefficient between SI and serum lactate was 0.273 (p=0.001). This correlation slightly increased at 24 hours post-admission, with a coefficient of 0.293 (p=0.001) (Table 5).

**Table 5:** Correlation of SI with serum lactate. The Pearson correlation coefficient (r) was used to assess the strength and direction of the linear relationship between lactate levels (at admission and 24 hours) and the SI. A positive r-value indicates a direct correlation. SI: shock index

Variables	Pearson correlation coefficient	p-Value
Lactate at admission	0.273	0.001
Lactate at 24 hours	0.293	0.001

## Discussion

In this study involving 124 patients with shock from various etiologies, hypovolemic shock emerged as the most prevalent type, affecting 61.3% (n=76) of the cohort. Septic shock accounted for 30.6% (n=38), while cardiogenic shock was observed in 8.1% (n=10) of the study population. Serum lactate levels were analyzed both at admission and 24 hours post-admission. The mean lactate level at admission was 4.65±3.83 mmol/L, which increased to 6.82±3.83 mmol/L at 24 hours. The overall mortality rate among study participants was 28.2%(n=35). Notably, patients who succumbed had significantly higher mean lactate levels both at admission (7.68 mmol/L) and at 24 hours (10.17 mmol/L) compared to survivors (3.46 mmol/L at admission and 5.56 mmol/L at 24 hours), indicating the prognostic value of elevated lactate in predicting mortality. Mechanical ventilation was required in 52.4% (n=65) of patients, and this requirement was significantly associated with elevated serum lactate levels at both time points. Receiver operating characteristic (ROC) curve analysis demonstrated that serum lactate levels at admission and 24 hours were strong predictors of the need for mechanical ventilation, with areas under the curve (AUC) of 0.73±0.04 and 0.66±0.05, respectively. Similarly, they were strong predictors of mortality, with AUCs of 0.765±0.049 and 0.848±0.042, respectively. These findings reinforce the utility of serial lactate measurements in early risk stratification and prognostication in patients with shock. Additionally, a weak positive correlation was observed between the shock index and serum lactate levels at both admission (r=0.273) and 24 hours post-admission (r=0.293), indicating a modest association between hemodynamic instability and lactate elevation.

In the present study, hyperlactatemia was observed at both admission and 24 hours in all shock patients, which is consistent with findings from previous studies [4,7-10,13,14]. This supports the role of lactate as a reliable marker of tissue hypoperfusion across various types of shock.

The overall mortality rate in our cohort was 28.2%, aligning closely with previous studies by Chaudhari and Agarwal and Tejaswini et al., who reported mortality rates of 23.5% in septic shock patients [15,16]. Our broader inclusion of multiple shock types enhances the applicability of our findings across a more heterogeneous clinical population. In line with prior research, our results reinforce that elevated lactate levels are robust predictors of mortality. This is further supported by our receiver operating characteristic (ROC) curve analysis, which revealed that lactate levels at admission and 24 hours had areas under the curve (AUCs) of 0.765±0.049 and 0.848±0.042, respectively, indicating good discriminatory ability. Comparable findings have been reported in the literature. Chaudhari and Agarwal demonstrated AUCs of 0.823 and 0.948 at admission and 24-hour lactate levels, respectively [15], while Tejaswini et al. observed AUCs of 0.80 and 0.92 for predicting 28-day mortality based on lactate levels at admission and 48 hours [16]. Similarly, Lee et al. found that lactate levels at admission and 6 hours were predictive of 30-day mortality in septic shock patients (AUCs of 0.612 and 0.720, respectively) [17]. These data collectively support the use of serial lactate monitoring as a dynamic tool for prognostication in shock patients.

Additionally, a systematic review and meta-analysis by Zhang and Xu, which synthesized data from 15 studies, further affirmed that hyperlactatemia is a common finding in patients with shock [18]. The review also demonstrated that higher lactate clearance is significantly associated with reduced mortality, highlighting the clinical relevance of both lactate magnitude and trajectory in guiding management decisions.

In our study, mean serum lactate levels increased at 24 hours post-admission in both survivors and non-survivors. This finding contrasts with previous studies by Chaudhari and Agarwal and Tejaswini et al., which reported a decline in lactate levels among survivors at 24 hours [15,16]. However, those studies focused exclusively on patients with septic shock. The unexpected rise in lactate among survivors in our cohort may be attributed to the inclusion of a heterogeneous shock population with varying etiologies, differences in underlying comorbidities, and individual variations in lactate clearance kinetics. Importantly, the rise in serum lactate at 24 hours was more pronounced in non-survivors compared to survivors, reinforcing its value as a prognostic indicator.

The requirement for mechanical ventilation was also significantly associated with higher lactate levels at both time points. This emphasizes that serum lactate, beyond its association with mortality, may have broader utility in anticipating clinical deterioration and identifying patients at risk of requiring advanced supportive care, such as mechanical ventilation.

We also examined the correlation between serum lactate and the shock index, a surrogate marker for hemodynamic compromise. A modest positive correlation was observed at both admission (r=0.273) and at 24 hours (r=0.293), suggesting that rising lactate levels may reflect the degree of hemodynamic instability. While the correlation is not strong, it supports the concept that lactate and shock index may provide complementary insights into shock severity. A study done by Berger et al. also found that shock index predicts hyperlactatemia and 28-day mortality in sepsis patients. This study showed that patients with a normal SI (less than 0.7) are 95% likely not to present with a high lactate level [19].

Importantly, this study expands upon prior literature by assessing the trajectory of lactate levels over 24 hours in a mixed population of shock patients, rather than focusing solely on a single measurement or a specific shock subtype. This approach enhances the clinical relevance of our findings and underscores the value of serial lactate assessments for dynamic risk stratification. Furthermore, our analysis of the relationship between lactate and the shock index adds a novel dimension to the understanding of lactate’s role in evaluating circulatory failure.

Strengths and limitations

This study has several strengths. The inclusion of patients with diverse shock etiologies improves the generalizability of our findings, and the analysis of serial lactate levels provides a more comprehensive view of lactate kinetics in critical illness. However, certain limitations must be acknowledged. First, the study was conducted at a single tertiary care center, which may limit the external validity and generalizability of the findings. Second, the overall sample size was relatively small compared to larger studies in this field, which may have limited the statistical power and precision of our estimates. This concern is underscored by the large standard deviations observed in lactate values, which may reflect variability due to the limited sample size. Third, the observed rise in lactate levels at 24 hours across all patient groups, including survivors, is an unexpected finding that may raise questions regarding the validity or presence of unmeasured confounding factors. Fourth, while inclusion of patients with diverse shock etiologies was intended to enhance generalizability, it also introduced heterogeneity in disease processes, reversibility, and baseline mortality risk factors that could confound the relationship between lactate dynamics and clinical outcomes. Additionally, we did not include other potentially relevant clinical or laboratory parameters, such as inflammatory biomarkers or measures of lactate clearance, which may have provided further insight. Finally, the lack of adjustment for key confounders such as age, baseline comorbidities (e.g., diabetes, chronic kidney disease), use of vasopressors, organ dysfunction scores (such as Sequential Organ Failure Assessment {SOFA} or Acute Physiology and Chronic Health Evaluation II {APACHE II}), and initial fluid resuscitation adequacy limits the ability to ascertain the independent predictive value of lactate in this cohort.

## Conclusions

This study highlights the prognostic significance of serum lactate levels measured at admission and 24 hours in patients with shock of various etiologies. Elevated lactate levels were strongly associated with increased mortality and the need for mechanical ventilation, underscoring their utility in early risk stratification. Although lactate levels rose in both survivors and non-survivors, the rise was significantly greater among non-survivors, reinforcing its role as a dynamic prognostic marker. The modest correlation between shock index and lactate further supports their complementary role in assessing circulatory compromise. While our findings align with prior research in septic shock, this study extends their relevance to a heterogeneous shock population in a real-world, resource-limited setting. However, the single-center design, small sample size, and absence of adjustment for confounders warrant cautious interpretation. Future larger, multi-center studies are needed to validate these findings and further explore the utility of serial lactate monitoring in diverse shock subtypes.
